# Correction: Survivorship and yield of a harvested population of Forsteronia glabrescens

**DOI:** 10.1371/journal.pone.0315020

**Published:** 2024-12-02

**Authors:** Demetrio Luis Guadagnin, Paulo Vinícius Fernandes Barradas

The following information is missing from the Acknowledgment statement: We would like to thank the Coordenação de Aperfeiçoamento de Pessoal de Nível Superior—Brasil (CAPES) for supporting this study.
