# Supplementary material for: Survivorship and yield of a harvested population of Forsteronia glabrescens
Source: PLoS One. 2022 Jun 8;17(6):e0268632. doi: 10.1371/journal.pone.0268632 (PMC9176754; doi:10.1371/journal.pone.0268632)
Supplement: Attachment — Submitted filename: PONE-D-21-21671 rebuttal letter 2.pdf [file pone.0268632.s003.pdf]

Below, we address each specific comment made by the referees, addressed in the revised manuscript "**Survivorship and yield of a harvested population of *Forsteronia glabrescens***" (PONE-D-21-21671 EMID bfb9dffccce607f0, R1).

<u>Responses to comments:</u>

Journal Requirements:

#1. Please review your reference list to ensure that it is complete and correct. If you have cited papers that have been retracted, please include the rationale for doing so in the manuscript text, or remove these references and replace them with relevant current references. Any changes to the reference list should be mentioned in the rebuttal letter that accompanies your revised manuscript. If you need to cite a retracted article, indicate the article's retracted status in the References list and also include a citation and full reference for the retraction notice.

→ We added five references to account for the issues raised by the referees. Two to detail information about the Kaingang people and their usage of NTFPs in South Brazil; one as proof of the old-growth condition of the studied forest stand; and two to justify the robustness of the design and results despite unequal sample sizes. The included references are:

Freitas, AEC. Mrur Jykre – A cultura do cipó: territorialidade Kaigang na margem leste do Lago Guaíba, Porto Alegre, RS. Doctoral dissertation. Universidade Federal do Rio Grande do Sul. 2005. Available from: https://www.lume.ufrgs.br/handle/10183/14922

Fortes, PHR. Indígenas na cidade: Uma análise histórica e etnográfica da presença Kaigang em Curitiba. Doctoral dissertation. Universidade Federal do Paraná. 2020. Available from: https://acervodigital.ufpr.br/handle/1884/69422

Ribeiro, CV. Preceituação ecológica para a preservação dos recursos naturais na região da Grande Porto Alegre. Porto Alegre: Fundação Zoobotânica do Rio Grande do Sul; 1976.

Maas CJM, Hox JJ. Sufficient Sample Sizes for Multilevel Modeling. Methodology. 2005;1:86–92. https://doi.org/10.1027/1614-2241.1.3.86

Zuur AF, Ieno EN, Elphick CS. A protocol for data exploration to avoid common statistical problems: Data exploration. Methods in Ecology and Evolution. 2010;1:3–14. https://doi.org/10.1111/j.2041-210X.2009.00001.x

Reviewers' comments:

Review Comments to the Author

Reviewer #3:

#2. This ms presents the results of an interesting, relevant and well conducted study on the sustainability of liana harvesting in Brazil. The experimental is appropriate, statistical analyses are sophisticated and correct, and results are interesting. I have some minor comments.

→ Thank you for the recognition of our effort and outcome.

#3. Figures. In Figures 2 and 3, the unit of y-axis is incomplete. Is this annual survival and growth?

→ Fig.2 shows three sets of bars representing different periods – the first two of six months and the last one of one year. Because of that, we specified the length of each set in the subtitles instead of in the y-axis. We rephrased the subtitle to make it clearer. New subtitle reads: "Fig 2. Survivorship of Forsteronia glabrescens in two harvesting periods of six-months and after one year, comparing stems unharvested and submitted to harvesting once and twice a year."

#4. I was confused by Figure 2: if for the first and second half-year period survival is expressed per half year, the numbers do not match, because a 65% survival in the semi-annual treatment in the first period could never lead to an ~80% survival for the full year. So my guess is that survival is annualized, but it's important to be clear about this, also in the text.

→ Thank you very much for finding that error in Fig. 2. We had pulled the wrong numbers when building Fig. 2 and apologize for that. The true figures of survival are 66% in the semi-annual

treatment in the first period, 73% the second period, and 53% for the whole year. We replaced Fig. 2 and also corrected the values mentioned in the text. All interpretations remain the same, since they were based on the correct figures.

#5. Negative radial growth. I was surprised to see negative radial growth rates. Are these measurement errors, or are they resulting from the fact that growth rate is based on the average diameter? In the latter case, I suggest to avoid confusion by calling this 'Change in mean stem diameter', instead of radial growth. IN any case, this needs to be better explained in the methods section.

→ Thank you for the suggestion. We accepted it and changed the mentions to radial growth in the table 1 and along the text. We agree it will help to avoid confusion. We also explained in Methods that we calculated and then analyzed mean values. In order to standardize the wording and be more precise, we are using "Change in stem length" as well, instead of "stem growth". See also issue #28.

#6. Textual comments:

Line 251, remove "does"?

→ Done.

#7. Line 261-262. Strange formulation; I suggest to change to: "Our results raise concerns about the sustainability of stem harvesting of our study species and we therefore call for continued monitoring of harvested populations from this species."

→ Done.

Reviewer #4: General considerations

#8. Dear authors and editor in chief, the manuscript (PONE-D-21-21671) investigated how the exploitation influences survivorship, growth and yield of Forsteronia glabrescens, a liana from subtropical South America. The article is well written and address an important subject for the conservation of forests: how management practices affect species. Furthermore, the conclusions are supported by the gathered data and the influences of the weaknesses (one location and one-year long study) are properly discussed.

Authors did an excellent job addressing the suggestions of the previous referees. Thus, I made my few comments and suggestions based on the second version of the manuscript, as you will find below.

→ *Thank you for the recognition of our effort and outcome*

Introduction

#9. It would be nice to see some lines quantifying the importance of harvesting the Forsteronia glabrescens for the Kaingang people. For instance, how many families depends upon this harvesting? Alternatively, how much money they can make by selling the handicrafts? These informations can give a better picture about the importance of this species to the people who manage it.

→ *We included a statement regarding the number of Kaingangs in South Brazil and the importance of crafts and NTFPs. Unfortunately, there are no good figures. Our previous work [ref. 26] is the only one providing economic figures of the exploitation of lianas by Kaingangs. We cited two unpublished thesis in order to provide some figures.*

Methods

#10. Please provide, if possible, any historic information about the forest used to install and evaluate the plots. For instance, is there any information about previous harvesting of lianas in the studied area?

→ We expanded the last sentence of the first paragraph of the Methods section, providing the figure that the old forest stand studied is out of direct human interference at least in the last 50 years.

#11. It is not clear to me how the authors compared the means of survivorship, radial growth and harvested length between treatments. For instance, survivorship in the control group is considered higher than the other treatments for the first period (lines 161-162), but based on what statistical test?

→ As stated in the Methods section, we used GLMM to analyze the data. Our statement about the significance of differences about treatments is based on the inclusion of the allocation of individuals in the experiment as an explanatory, categorical variable in the model (see page 6, lines 127-129 in the checked new version).

Results

#12. Figures 2 to 4 are not standing alone. What is the statistic depicted in the error bars? Confidence interval, standard deviation…? It seems to me that comparisons between means were made based on the error bars, but it remains unclear what statistic is depicted there and how it was estimated. Please provide this information in methods section.

→ Done. We rephrased the captions of figures 2, 3 and 4, and included the statement that the values represent means ± standard errors.

#13. The scales of Fig 2 and 4 are wrong, because it varies from zero to one. If it is a percentage, it should vary from zero to 100.

→ We are providing a new figure correcting the scale, as well as showing the data in a slightly different way to make it more clear – we are showing the change in the mean stem length of each period as well as the accumulated values at the end of the study. See also issues #26, #30, and #31.

#14. Please use points as decimal separator.

→ *Checked.*

Reviewer #5:

#15. I believed the authors addressed properly the questions of the Reviewer 1. I read the manuscript again and found a few things that still need to be addressed. Most of them are related to unclear or confusing sentences and statements and are of a simple solution. The paper focus on a very interesting subject, the impact of vines extraction on the vine population structure, survivorship, and yield. The authors made an experiment simulating two extraction intensities (similar to those practiced by traditional human exploitation of the vines) and evaluated the results for the vine population. Their results showed that both treatments (extraction once a year and extraction twice a year) do not allow enough time to population recover, resulting in a lower yield and reduction of the population. The results and conclusions seem robust. Although, in my opinion, the number and size of the plots used as replicas could both be larger, the previous reviewers did not see any problem there and I agree that the results are solid. A larger sample possibly only leads to other significant results not found here, but certainly would not result in denying the significant results presented in the paper.

→ *Thank you for the recognition of our effort and outcome.*

Minor comments:

#16. Ln 51-52, 55-56, 224-225: When you say “compensatory and additive mortality”, I guess you mean that the recruitment after exploitation can have a compensatory or additive effect on the population. If so, it is not mortality that is compensatory or additive, but the recruitment as a response to the higher mortality generated by the exploitation.

→ *We prefer to keep the vernacular wording of the concept – compensatory and additive mortality*

– as defined in the mentioned literature. Hoping to clarify the idea, we rephrased the statements in lines 51-52, 55-56 to add the term "recruitment". This term is already used in lines 224-225, so we left them unchanged.

#17. Ln 58-60: Obscure sentence. What do you mean? Reduction of the population?

→ We rephrased the statement, focusing on the trade-off between recruitment and reproduction.

#18. Ln 100-102: The different number of replicas by treatments could be a problem. I saw that the first reviewer also pointed to this problem. However, you are right; the statistical methods adopted are robust to overcome this shortcoming. However, it is hard to understand why you did that, so you need to justify this decision in the text.

→ We included a statement about the unbalanced design in the paragraph commenting the weaknesses, in the Discussion. One plot of the annual harvest treatment had some labels lost or removed, probably by fauna, and was excluded from analyses.

#19. Ln 134-136: Why is this in italic?

→ This was an error probably at the pdf building. The original text is not in italic.

#20. Ln 158-159: You need to tell the readers what it is DEC/14 and DEC/15.

→ We were unable to find this issue. Probably a problem only in the version submitted to the referee.

#21. Ln 162-163: Rewrite "About all the mortality after the initial harvest occurred in this period."

→ Done.

#22. Ln 163-164: These results contradict the graphic in Fig. 2. There, the annual harvest had less than 60% of survivorship. The semi-annual has survivorship higher than 72% after 1 yr.

→ Corrected. Thank you for highlighting this error. We apologize for that. Actually, the numbers in the text were wrong. The correct values, as shown in Fig. 2, are 80.70% for the control group, 55.81% in the annual harvest and 53.19% in the semi-annual harvest.

#23. Ln 164-165: Looking at the table, I cannot see this result “The cutting of stems reduced the survivorship in all treatments and periods.” The only thing I can see there is that the stem cutting affected the survivorship in the first period and, by consequence, in the whole period. No distinction is made for treatments.

→ We agree with the referee’s interpretation. Survivorship was affected, either positively or negatively, in all periods, but by different factors. Stem cutting only affected survivorship in the first period. We modified the statement about this result. We checked this subject in the discussion, but no conclusion needed to be reformulated.

#24. Ln 166-167: But the maximum tree diameter had a negative relationship with survivorship. Do not omit results.

→ Included. Although we had initially opted to not highlight this result in the text of Results section, we have discussed the issue in the Discussion section.

#25. Ln 168: Table 1: This is hard to read. Some numbers seem to be out of place. Fix the table.

→ Fixed.

#26. Ln 170-171: Fig. 2. You say that the y axis is %, but it is not. You have to multiply the number by 100 to get the %.

→ Done. See also issues #12, #30, and #31.

#27. Ln 173: Checking the figure, the control group reduced in diameter, mainly during the first period.

→ *We included a statement in the results commenting that the control group showed minor variations in stem diameter along the year (maximum of 5%). These variations are caused by a few deaths and births (sprouts) along the period. We regard them as not relevant and did not discuss the issue.*

#28. Ln 176: "The radial growth increased with the cutting of stems" happened only in the first period.

→ *We included this detail in the corresponding phrase. See also issue #5.*

#29. Ln 189 Does "while the treatment groups measured 199.9 ± 130.8 cm" refer to both treatments together. Be clear about that.

→ *Stated explicitly, as asked.*

#30. Ln 190-191: "The growth in length of the harvested groups after one year was in average 40% lower than their initial length." Where is this result shown?

→ *This result can be depicted from Fig. 4. We rebuilt this figure, changing the values shown from percentage change to percentage to facilitate the interpretation. We opted to not include another table to keep the manuscript short. See also issues #13 and #26.*

#31. Ln 191-192: I could not find this result in the table.

→ *This result can also be depicted from Fig. 4. See #30.*

#32. Ln 209: Exchange "Although" for "However".

→ *Done. Thank you.*

#33. Ln 210-211: “Other cases of exploitation of perennial, understory NTFP showed comparable results to ours”. Too vague, how comparable? Maybe, "similar", but you should give the readers some parameters to conclude that.

→ We expanded the statement.

#34. Ln 211-216: You are repeating results presented previously. Right now you need to offer the reader some insights.

→ Here we are highlighting the proofs for the conclusion that the population of F. grabrescens under-compensates the exploitation of stems at the experimented conditions. We included another phrase stating this. We believe that is essential to be explicit about all the effects of the harvesting on survivorship and stem diameter and length.

#35. Ln 220-222: So, are you saying that the studied plants are investing more in below-ground parts? Can you prove it or you are only speculating? Your results show that the studied plant is not recovering enough in a single year, but saying that the plants are relocating energy to underground structures is a little too speculative, although an interesting point that could be suggested for further investigation.

→ We believe we are being clear that the investment in bellow-ground organs is a discovery of others (reference [10]), under natural situations that, for the fate of the plant, are similar to a human exploitation (predation). Here we are pointing to an under-investigated subject in hope of stimulating other studies.

#36. Ln 234: The sentence “The density of F. glabrescens is similar to that found in other studies” is lost here.

→ We deleted the statement. It is just a minor comment.

#37. Ln 242-244: Expand this idea. It seems an interesting one.

→ Expanded. We added a comment about the probable population outcome when the relationship is facultative and services, instead o food, are demanded by the parasite, as the case of lianas and trees.

While revising your submission, please upload your figure files to the Preflight Analysis and Conversion Engine (PACE) digital diagnostic tool, https://pacev2.apexcovantage.com/.
