# Supplementary material for: Survivorship and yield of a harvested population of Forsteronia glabrescens
Source: PLoS One. 2022 Jun 8;17(6):e0268632. doi: 10.1371/journal.pone.0268632 (PMC9176754; doi:10.1371/journal.pone.0268632)
Supplement: Attachment — Submitted filename: PONE-D-21-21671 rebuttal letter 3.pdf [file pone.0268632.s004.pdf]

Below, we address the specific comments made upon the second version of the manuscript "**Survivorship and yield of a harvested population of *Forsteronia glabrescens***"

<u>Responses to comments:</u>

After careful consideration, we feel that it has merit but does not fully meet PLOS ONE's publication criteria as it currently stands. Therefore, we invite you to submit a revised version of the manuscript that addresses the points raised during the review process.
Thank you for completing another round of revisions. I appreciate your attention to the reviewers' comments. I believe the revised text is suitable for publication.
→ *We are very happy to have reached the journal standards.*

but there is a problem with the figures: what you uploaded as the new Fig. 2 was actually meant to replace Fig. 3. Consequently, there is no figure depicting survivorship (the intended subject of Fig. 2). This is an easy fix -- simply upload the correct versions of Figs. 2 and 3. Furthermore, you might change the legend labels in Fig. 1 to "Dec 2014" and Dec 2015" for added clarity, but this is a relatively minor detail.
→ *We updated the figures as requested.*
