# Supplementary material for: Survivorship and yield of a harvested population of Forsteronia glabrescens
Source: PLoS One. 2022 Jun 8;17(6):e0268632. doi: 10.1371/journal.pone.0268632 (PMC9176754; doi:10.1371/journal.pone.0268632)
Supplement: Attachment — Submitted filename: PONE-D-21-21671 Response to Reviewers.odt [file pone.0268632.s002.odt]

Below, we address each specific comment made by the referees, addressed in the revised manuscript “Survivorship and yield of a harvested population of Forsteronia glabrescens” (PONE-D-21-21671 EMID bfb9dffccce607f0).

Responses to comments:

Journal Requirements:
When submitting your revision, we need you to address these additional requirements.

1. Please ensure that your manuscript meets PLOS ONE's style requirements, including those for file naming. The PLOS ONE style templates can be found at
https://journals.plos.org/plosone/s/file?id=wjVg/PLOSOne_formatting_sample_main_body.pdf and https://journals.plos.org/plosone/s/file?id=ba62/PLOSOne_formatting_sample_title_authors_affiliations.pdf.
→ We corrected the style of authorship, titles and fig captions, as well as the figure’s formats, as requested.

2. In your Methods section, please provide additional information regarding the permits you obtained for the work. Please ensure you have included the full name of the authority that approved the field site access and, if no permits were required, a brief statement explaining why.
→ We included the statement that “ The field research was authorized by the Secretaria Municipal do Meio Ambiente da Prefeitura Municipal de Porto Alegre under the Permit no. 308/07 “ in Methods, lines 96-97.

3. We note that you have indicated that data from this study are available upon request. PLOS only allows data to be available upon request if there are legal or ethical restrictions on sharing data publicly. For more information on unacceptable data access restrictions, please see http://journals.plos.org/plosone/s/data-availability#loc-unacceptable-data-access-restrictions.
In your revised cover letter, please address the following prompts:
a) If there are ethical or legal restrictions on sharing a de-identified data set, please explain them in detail (e.g., data contain potentially sensitive information, data are owned by a third-party organization, etc.) and who has imposed them (e.g., an ethics committee). Please also provide contact information for a data access committee, ethics committee, or other institutional body to which data requests may be sent.
b) If there are no restrictions, please upload the minimal anonymized data set necessary to replicate your study findings as either Supporting Information files or to a stable, public repository and provide us with the relevant URLs, DOIs, or accession numbers. For a list of acceptable repositories, please see http://journals.plos.org/plosone/s/data-availability#loc-recommended-repositories.
We will update your Data Availability statement on your behalf to reflect the information you provide.
→ We included a table with crude field data as Supporting Information. See answers #7 and #13

4. Please include your full ethics statement in the ‘Methods’ section of your manuscript file. In your statement, please include the full name of the IRB or ethics committee who approved or waived your study, as well as whether or not you obtained informed written or verbal consent. If consent was waived for your study, please include this information in your statement as well.
→ We included the statement that “The field research was authorized by the Secretaria Municipal do Meio Ambiente da Prefeitura Municipal de Porto Alegre under the Permit no. 308/07 “ in Methods, lines 96-97. See answer to question #2, above. 

5. Please review your reference list to ensure that it is complete and correct. If you have cited papers that have been retracted, please include the rationale for doing so in the manuscript text, or remove these references and replace them with relevant current references. Any changes to the reference list should be mentioned in the rebuttal letter that accompanies your revised manuscript. If you need to cite a retracted article, indicate the article’s retracted status in the References list and also include a citation and full reference for the retraction notice.
→ We reviewed the references and in-text citation thoroughly according to the most recent guidelines
→ We added ten new references, attending questions #15 and #19, bellow:
1. Kor L, Homewood K, Dawson TP, Diazgranados M. Sustainability of wild plant use in the Andean Community of South America. Ambio. 2021;50:1681–1697. https://doi.org/10.1007/s13280-021-01529-7
2. Cunningham, AB, Brinckmann JA, Harter DEV. From forest to pharmacy: Should we be depressed about a sustainable Griffonia simplicifolia (Fabaceae) seed supply chain?,Journal of Ethnopharmacology, 2021;278:114202, https://doi.org/10.1016/j.jep.2021.114202
3. Zhou S, Lou Y-R, Tzin V, Jander G. Alteration of Plant Primary Metabolism in Response to Insect Herbivory. Plant Physiology. 2015;169:1488–1498. https://doi.org/10.1104/pp.15.01405
4. Li C, Barclay H, Roitberg B, Lalonde R. Ecology and Prediction of Compensatory Growth: From Theory to Application in Forestry. Frontiers in Plant Science. 2021;12:1352. https://doi.org/10.3389/fpls.2021.655417
5. Zhang T, Yu L, Man Y, Yan Q. Effects of harvest intensity on the marketable organ yield, growth and reproduction of non-timber forest products (NTFPs): implication for conservation and sustainable utilization of NTFPs.Forest Ecosystems. 2021;8:56. https://doi.org/10.1186/s40663-021-00332-w
6. da Cunha Vargas B, Grombone-Guaratini MT, Morellato LPC. Lianas research in the Neotropics: overview, interaction with trees, and future perspectives. Trees;2021;35: 333–345. https://doi.org/10.1007/s00468-020-02056-w
7. Mori H, Ueno S, Kamijo T, Tsumura Y, Masaki T. Interspecific variation in clonality in temperate lianas revealed by genetic analysis: Do clonal proliferation processes differ among lianas? Plant Species Biology. 2021;36:578–588. https://doi.org/10.1111/1442-1984.12348
8. Estrada-Villegas S, Hall JS, van Breugel M, Schnitzer SA. Lianas reduce biomass accumulation in early successional tropical forests. Ecology. 2020;101:e02989. https://doi.org/10.1002/ecy.2989
9. Lee ED, Kempes CP, West GB. Growth, death, and resource competition in sessile organisms. Proceedings of the National Academy of Sciences. 2021;118:e2020424118. https://doi.org/10.1073/pnas.2020424118
10. Ceballos SJ, Malizia A. Liana density declined and basal area increased over 12 y in a subtropical montane forest in Argentina. Journal of Tropical Ecology. 2017;33:241–248. https://doi.org/10.1017/S0266467417000153

Additional Editor Comments (if provided):

6. The paper present some interesting results on population of Forsteronia glabrescens and it is of itnerest for the sustainable managment of this species. There are some comments by the two referies that should be taken into consideration for the authors, as they can improve the quality of the paper.
→ Thank you for the recognition of our effort.

7. You should specify if the data is included in any public repository as you mention that the data is accessible without restrictions.
→ We included a table with crude field data as Supporting Information. See answers #3 and #13.

8. Please, check the final part of the conclusion (Either larger areas, lower pressures, or longer resting periods are necessary to exploit F. glabrescens sustainably) as it seems that this conclusion is not directly extracted from your data.
→ Thank you for noting that. We made a minor change in this statement (added words in bold), which we judge are enough to clarify our idea: “Our work demonstrates that the exploitation of F. glabrescens in southern Brazil, either at six months or one year intervals, leads to increased mortality rate, reduced mean size of ramnets, yields smaller than expected in previously unharvested areas, and a risk of even smaller yields if the exploitation goes on continuously. Either lower pressures over large areas or longer resting periods may be necessary to exploit F. glabrescens sustainably.” (See lines 270-274 in the annotated version). We understand that this conclusion follows from the results we got. The traditional practice of harvesting lianas at six months or one year intervals were not enough for the standing crop to recover, as demonstrated. Since the species reproduces from underground sprouts and ramets are exploited, lower pressure (less ramets cut per plant) over large areas could lead to the same desired crop while allowing for the plants to recover. We discussed the self-thining hypothesis as a potential explanation. Furthermore, longer resting periods are certainly another efficient alternative, even though we still don’t know how long this resting time should be at different pressures.


9. I also think that your study is rectricted to one location, and therefore you should mention any caveats to extrapolate your results to the management of the species in general.
→ We recognize this caveat and consigned a whole paragraph to deal with it. We made minor changes in the last statements of that paragraph (here in bold), recognizing that our results are confident at least to “the Atlantic, secondary forests of southern Brazil where most of the exploitation occurs. We added that we are confident that “Our results are also enough to rise concern for the need of careful monitoring while exploring non-timber forest products”. (see lines 267-269 in the annotated version).We hope this changes will be enough as a recognition, at the same time, of caveats and reaches of our job.

10. Please check in line 144 after packages if the name of one package is missing before the first reference.
→ Corrected. We added the name of the package – bbmle.

Reviewers' comments:

Reviewer's Responses to Questions
Comments to the Author

11. Is the manuscript technically sound, and do the data support the conclusions?
The manuscript must describe a technically sound piece of scientific research with data that supports the conclusions. Experiments must have been conducted rigorously, with appropriate controls, replication, and sample sizes. The conclusions must be drawn appropriately based on the data presented. 
Reviewer #1: Yes
Reviewer #2: Yes
→ Thank you.
 
12. Has the statistical analysis been performed appropriately and rigorously? 
Reviewer #1: Yes
Reviewer #2: Yes
→ Thank you.
 
13. Have the authors made all data underlying the findings in their manuscript fully available?
The PLOS Data policy requires authors to make all data underlying the findings described in their manuscript fully available without restriction, with rare exception (please refer to the Data Availability Statement in the manuscript PDF file). The data should be provided as part of the manuscript or its supporting information, or deposited to a public repository. For example, in addition to summary statistics, the data points behind means, medians and variance measures should be available. If there are restrictions on publicly sharing data—e.g. participant privacy or use of data from a third party—those must be specified.
Reviewer #1: Yes
Reviewer #2: No
→ We included a table with crude field data as Supporting Information. See answers #3 and #7

14. Is the manuscript presented in an intelligible fashion and written in standard English?
PLOS ONE does not copyedit accepted manuscripts, so the language in submitted articles must be clear, correct, and unambiguous. Any typographical or grammatical errors should be corrected at revision, so please note any specific errors here.
Reviewer #1: Yes
Reviewer #2: Yes
→ Thank you.
 
Review Comments to the Author
Please use the space provided to explain your answers to the questions above. You may also include additional comments for the author, including concerns about dual publication, research ethics, or publication ethics. (Please upload your review as an attachment if it exceeds 20,000 characters)

15. Reviewer #1: The Manuscript presents important elements to guide sustainable extractivism.
References are out of date
→ We did our best to review the most recent advancements in subjects relates with our rationale about the ecological effects of the exploitation of NTFP in general, and of lianas in particular. This is a poorly investigated subject. We did find a few interesting examples and an interesting new focus for the self-thinning principle. Based on the new references we updated the second and third paragraphs of the discussion section. See also the answer to questions #5, above, and #19, bellow.

16. Uneven number of parcels could not have affected the results? what was the authors' strategy to resolve this situation?
→ GLMM is robust to departures from common assumptions of Anova, as it uses a weighted mean instead of a grand mean. Having unbalanced groups has little influence on multilevel ML provided it is not really strong (Maas & Hox 2005 doi: 10.1027/1614-1881.1.3.86; Zuur et al 2010 doi: 10.1111/j.2041-210X.2009.00001.x). The major effect of unbalanced design is heterogeneity of variances. We checked heterogeneity as recommended by Grueber et al 2011 doi: 10.1111/j.1420-9101.2010.02210.x. VIF values were low. Power problems could became important in the case of strong unequal variances which was not the case. We thus understand that this is not a major issue.

Reviewer #2:
The manuscript “Survivorship and yield of a harvested population of Forsteronia glabrescens” submitted to Plos One describes the effect of harvesting on survivorship of a liana species in forest area of Brazil. It is well written, the ideas are clear, and the interest part is that encompass several subjects (ethnobotany, ecology, and forest management). The following observations may improve this manuscript:

17. First paragraph in the Introduction is too long. I suggest the authors to divide the ideas in two shorter paragraphs.
→ Done.

18. The second paragraph introduces the idea that the population of some liana species are declining due to overexploitation. It would be necessary to add a sentence about increasing liana abundance in the last decades:
Schnitzer, S. A., & Bongers, F. (2011). Increasing liana abundance and biomass in tropical forests: emerging patterns and putative mechanisms. Ecology letters, 14(4), 397-406.
Ingwell, L. L., Joseph Wright, S., Becklund, K. K., Hubbell, S. P., & Schnitzer, S. A. (2010). The impact of lianas on 10 years of tree growth and mortality on Barro Colorado Island, Panama. Journal of Ecology, 98(4), 879-887.
→ We addressed this issue in the statement “The high growth rates of some species, and the rapid colonization of forest edges and early successional phases cause them to be abundant elements in secondary forests and altered fragments [16, 17]. Many lianas have great capacity of regenerating after damage, so that a new branch quickly replaces a damaged branch [18]”. Schnitzer & Bongers, (2011) was already cited. We added to new references on the subject – da Cunha Vargas et al (2021) and Estrada-Villegas et al 2020. We added ‘increase’ in the statement that “Despite attributes favouring their increase in abundance in primary and secondary forests [16-18]. We didn’t want to expand to much the comments because the increase in abundance is not the focus of our research. See also question #5.

19. I recommend adding general information about liana demography in the Introduction. For example, relationships and trade-offs between growth and mortality, and shade-tolerance. Some papers on this regard:
→ We added more information about the self-thining effect, related with this subject. This is an interesting subject, since exploitation alleviates density, but the self-thining effect has not yet been addressed directly in exploitation studies. Gianoli et al (2010) was already cited. We added Ceballos and Malizia (2017). See also question #5.

20. It would be necessary to explain why these variables were selected: tree density, mean and maximum tree diameter at breast height (DBH), and tree DBH standard deviation, and how these variables are related to this particular liana species. There are some information on the literature? For example, tree DBH standard deviation represents the availability of trees of different sizes to climb to the canopy?
→ We expanded the statement declaring the reason to measure these variables in the methods section: “We used four variables describing the vegetation within each plot as random effects in order to control for the effect of potential differences in the forest stands on the abundance and fate of lianas and the availability of support trees and canopy cover: tree density, mean and maximum tree diameter at breast height (DBH), and tree DBH standard deviation. These DBH measures can account for potential effects of the variability in the availability of support trees and in the canopy cover, known to affect the abundance and growth of lianas [28, 29]”. We do not have predictions or hypotheses related with the random effects.

21. What was the minimum diameter for a liana to be considered? This information is needed in Methods.
→ We added in the methods that “We excluded from counting and harvesting young sprouts with length <1.0 m or diameter <0.2 mm, because they are not suitable for making handicrafts [26]”.

22. In Results change DAP by DBH.
→ done

23. In line 165, remove “The”.
→ done

24. Improve the quality of Figure 4.
→ done

25. Vines and ethnobotany are keywords but never mentioned in the text.
→ we selected synonyms as keywords in order to improve the chances of the paper reaching the interested audience. We preferred to standardize the words in the text.

26. It would be useful to add a Discussion on methodological issues involve in this study. Particularly, about the differences between doing this study in an area not exploited before (a relatively protected area in late succession) and not in areas that were harvested several times in the past. This probably affected the Results.
→ We declared that our objective was to reproduce “experimentally, under field conditions, the stem harvesting practised by the Kaingang natives to test if survivorship and yield differ between exploited and unexploited plots”. We rephrased the first sentence of the discussion in order to be more explicit about the reach of our results: “In this work we demonstrate that, compared with unexploited plots, the survivorship of Forsteronia glabrescens decreases in harvested plots, …”. The whole first paragraph focus on the comparison between exploited and unexploited stands.
